# The Complexity of the Tumor Microenvironment in Hepatocellular Carcinoma and Emerging Therapeutic Developments

**DOI:** 10.3390/jcm12237469

**Published:** 2023-12-02

**Authors:** Antonella Argentiero, Antonella Delvecchio, Rossella Fasano, Alessandro Andriano, Ingrid Catalina Caradonna, Riccardo Memeo, Vanessa Desantis

**Affiliations:** 1 Istituto Tumori “Giovanni Paolo II”, 70124 Bari, Italy; r.fasano@oncologico.bari.it; 2 Unit of Hepato-Biliary and Pancreatic Surgery, “F. Miulli” General Hospital, 70021 Bari, Italy; antodel88@libero.it (A.D.); drmemeo@yahoo.it (R.M.); 3 Department of Precision and Regenerative Medicine and Ionian Area, Pharmacology Section, University of Bari Aldo Moro Medical School, 70124 Bari, Italy; a.andriano2@studenti.uniba.it (A.A.); i.caradonna1@studenti.uniba.it (I.C.C.); vanessa.desantis@uniba.it (V.D.)

**Keywords:** hepatocellular carcinoma, tumor microenvironment, immunotherapy, targeted therapy, biomarkers, BCLC staging and classification

## Abstract

This review explores various aspects of the HCC TME, including both cellular and non-cellular components, to elucidate their roles in tumor development and progression. Specifically, it highlights the significance of cancer-associated fibroblasts (CAFs) and their contributions to tumor progression, angiogenesis, immune suppression, and therapeutic resistance. Moreover, this review emphasizes the role of immune cells, such as tumor-associated macrophages (TAMs), myeloid-derived suppressor cells (MDSCs), and regulatory T-cells (Tregs), in shaping the immunosuppressive microenvironment that promotes tumor growth and immune evasion. Furthermore, we also focused only on the non-cellular components of the HCC TME, including the extracellular matrix (ECM) and the role of hypoxia-induced angiogenesis. Alterations in the composition of ECM and stiffness have been implicated in tumor invasion and metastasis, while hypoxia-driven angiogenesis promotes tumor growth and metastatic spread. The molecular mechanisms underlying these processes, including the activation of hypoxia-inducible factors (HIFs) and vascular endothelial growth factor (VEGF) signaling, are also discussed. In addition to elucidating the complex TME of HCC, this review focuses on emerging therapeutic strategies that target the TME. It highlights the potential of second-line treatments, such as regorafenib, cabozantinib, and ramucirumab, in improving overall survival for advanced HCC patients who have progressed on or were intolerant to first-line therapy. Furthermore, this review explores the implications of the Barcelona Clinic Liver Cancer (BCLC) staging and classification system in guiding HCC management decisions. The BCLC system, which incorporates tumor stage, liver function, and performance status, provides a framework for treatment stratification and prognosis prediction in HCC patients. The insights gained from this review contribute to the development of novel therapeutic interventions and personalized treatment approaches for HCC patients, ultimately improving clinical outcomes in this challenging disease.

## 1. Introduction

Hepatocellular carcinoma (HCC) is the leading type of primary liver cancer and a significant global health burden. It ranks as the third leading cause of cancer-related deaths worldwide, with its incidence and mortality rates on the rise [1]. The increasing prevalence of HCC can be attributed to various factors, including the growing prevalence of chronic liver diseases, such as cirrhosis, hepatitis B and C infections, and nonalcoholic fatty liver disease (NAFLD) [2].

In the management of HCC, the Barcelona Clinic Liver Cancer (BCLC) staging and classification system has emerged as a widely used framework. This system takes into account tumor characteristics, liver function, and performance status to stratify patients into different stages (0 to D) and assign appropriate treatment strategies [3]. Patients with early-stage HCC (Stage 0 to A) have up to three nodules, each less than 3 cm, and are generally candidates for potentially curative therapies, such as surgical resection, liver transplantation, or local ablation [2]. Transarterial chemoembolization (TACE) is often recommended for intermediate-stage patients (Stage B), aka patients with multinodular HCC without vascular invasion or extrahepatic spread. Advanced-stage HCC (Stage C) patients are candidates for systemic therapies, such as molecular targeted therapies or immunotherapy. Sorafenib, as a multikinase inhibitor, is the first-line systemic therapy approved for advanced HCC and has shown modest survival benefits [4]. Lenvatinib, another targeted therapy, has also been approved as a first-line treatment option for unresectable HCC [5]. Unfortunately, the BCLC system does not consider molecular subtypes or genetic alterations that can impact prognosis and treatment response. Efforts are underway to integrate molecular profiling and genetic information into the BCLC system to improve its prognostic accuracy and treatment individualization [2]. Furthermore, it is important to recognize that the tumor microenvironment (TME) in HCC plays a critical role in tumor growth, invasion, metastasis, and therapeutic resistance.

The TME consists of a complex network of cellular and non-cellular components that interact dynamically to shape the behavior and progression of tumors. Understanding the essential components of the HCC TME and their roles in tumor progression is crucial for developing effective therapeutic strategies. This review aims to provide a comprehensive overview of the HCC TME, exploring both the cellular and non-cellular components and their contributions to tumor progression.

Moreover, the review delves into emerging therapeutic strategies that target the HCC TME. These strategies include second-line treatments, which have shown promising clinical benefits in patients who progressed or were intolerant to first-line therapy. Additionally, this review examines the utility of blood biomarkers in HCC diagnosis and surveillance, as well as their potential to enhance predictive capabilities for HCC recurrence and overall survival. Furthermore, it discusses the evolving role of immune checkpoint inhibitors (ICIs) [6,7,8] and BRAF-targeted therapies in HCC treatment, highlighting their potential benefits and challenges in this context [9,10]. The prognostic and predictive factors associated with HCC patients treated with sorafenib, a first-line systemic therapy for advanced HCC, are also explored. By understanding these factors, clinicians can better tailor treatment strategies and predict patient outcomes [11,12].

Lastly, this review provides an overview of the BCLC staging and classification system and its implications for treatment decisions, prognosis, and survival outcomes in HCC. Recognizing the importance of accurately stratifying patients based on their disease stage, liver function, and performance status is critical for selecting the most appropriate treatment approach and optimizing patient outcomes.

In summary, this comprehensive review aims to shed light on the essential components of the HCC TME, which are emerging therapeutic strategies that target the TME, and the implications of the BCLC staging and classification system. By deepening our understanding of the TME and its interplay with HCC, we can pave the way for the development of more effective and personalized treatment approaches for this devastating disease.

## 2. Tumor Microenvironment in HCC

### 2.1. Cellular Components

#### 2.1.1. Cancer-Associated Fibroblasts (CAFs)

Cancer-associated fibroblasts (CAFs) are the most abundant cell type in the HCC tumor microenvironment (TME) and play a crucial role in tumor progression and metastasis. CAFs are activated fibroblasts that have acquired distinct characteristics and functions in response to signals from cancer cells and the TME. They secrete various factors, including growth factors, cytokines, and extracellular matrix (ECM) proteins, which promote tumor cell proliferation, angiogenesis, immune suppression, and therapeutic resistance in HCC [13,14,15]. CAFs contribute to the remodeling of the ECM, creating a supportive niche for tumor growth and invasion [13]. Moreover, CAFs interact with other cell types within the TME, such as immune cells and endothelial cells, through paracrine signaling and direct cell–cell contact, further facilitating tumor progression and metastasis [13,14]. Finally, they play the following role in drug resistance: CAF-derived and secreted phosphoprotein 1 (SPP1) enhances tyrosine-kinase inhibitor resistance by activating alternative oncogenic signals and promoting epithelial-to-mesenchymal transition. The plasma SPP1 level represents a potential biomarker for sorafenib/lenvatinib treatment response prediction [16].

#### 2.1.2. Immune Cells

The immune response within the HCC TME is dysregulated, leading to immune evasion and tumor progression. Various immune cell populations have been identified in the HCC TME, including tumor-associated macrophages (TAMs), myeloid-derived suppressor cells (MDSCs), and regulatory T-cells (Tregs). TAMs, a type of macrophage, are key regulators of the immune response in HCC. They exhibit a distinct polarization toward an M2-like phenotype, characterized by the secretion of anti-inflammatory cytokines and growth factors, such as interleukin-10 (IL-10) and transforming growth factor-beta (TGF-β), promoting angiogenesis, tissue remodeling, and immune suppression [17]. TAMs also inhibit T-cell activation and function through the secretion of inhibitory molecules, including programmed death-ligand 1 (PD-L1), thereby contributing to immune evasion in HCC [18].

MDSCs represent another immune cell population that contributes to immune suppression in HCC. They are a heterogeneous population of immature myeloid cells with immunosuppressive properties. MDSCs inhibit T-cell responses through various mechanisms, such as the production of arginase-1 and inducible nitric oxide synthase (iNOS), leading to the depletion of essential nutrients and the generation of reactive oxygen species (ROS) [19]. This inhibitory environment hampers effective anti-tumor immune responses and promotes tumor progression in HCC.

**Figure 1:**
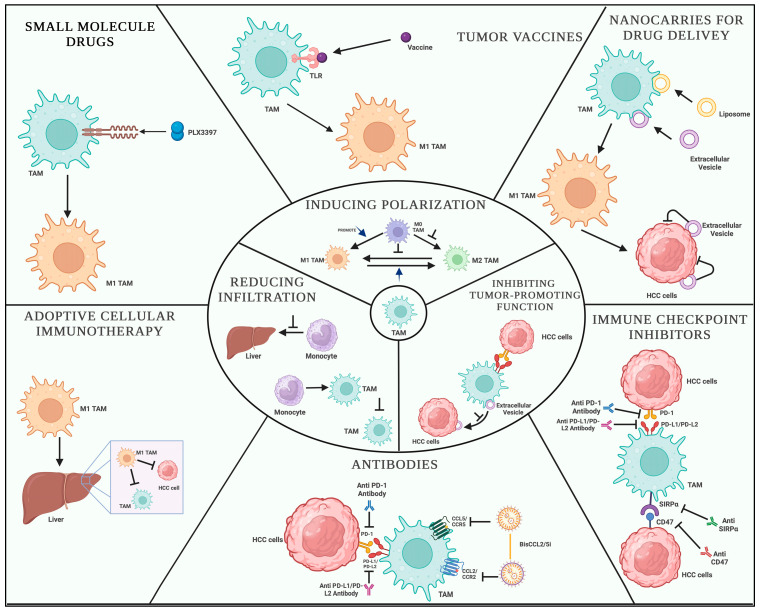
Immune cells in the tumor microenvironment (TME).

Tregs are a specialized subset of CD4+ T cells known for their immunosuppressive functions. They play a critical role in maintaining immune homeostasis and preventing excessive immune responses. In the HCC TME, Tregs accumulate and exert their suppressive effects by inhibiting effector T-cell responses and promoting tolerance to tumor antigens [19]. The presence of Tregs in the TME has been associated with poor prognosis and reduced survival in HCC patients (Figure 1).

### 2.2. Non-Cellular Components

#### 2.2.1. Extracellular Matrix (ECM)

The ECM is a complex network of proteins and polysaccharides that provides structural and biochemical support to cells within the TME. In HCC, the ECM undergoes dynamic changes that promote tumor growth, invasion, and metastasis. Alterations in the composition of ECM, remodeling enzymes, and stiffness affect cellular behaviors, such as cell adhesion, migration, and signaling pathways that are involved in tumor progression [20]. The dysregulated ECM in HCC contributes to the invasive and metastatic behavior of tumor cells by providing physical scaffolding and modulating cellular signaling events. Additionally, the abnormal ECM can create a barrier that limits the penetration and efficacy of therapeutic agents.

#### 2.2.2. Hypoxia and Angiogenesis

Hypoxia, characterized by an inadequate oxygen supply, is a hallmark of the HCC TME. It arises due to the rapid proliferation of tumor cells, insufficient vascularization, and the abnormal architecture of tumor blood vessels. Under hypoxic conditions, hypoxia-inducible factors (HIFs), particularly HIF-1α and HIF-2α, are stabilized and translocated to the nucleus, where they activate the expression of genes involved in angiogenesis, glycolysis, and cell survival [21]. In HCC, hypoxia-induced HIF activation promotes the secretion of pro-angiogenic factors, including vascular endothelial growth factor (VEGF), platelet-derived growth factor (PDGF), and angiopoietin-2 (Ang-2), which stimulate the formation of new blood vessels and the recruitment of endothelial cells [22]. This hypoxia-driven angiogenic response supports tumor growth, provides nutrients and oxygen to tumor cells, and facilitates metastasis by promoting the formation of abnormal and leaky blood vessels.

Overall, the cellular and non-cellular components of the HCC TME, including cancer-associated fibroblasts, immune cells, the extracellular matrix, hypoxia, and angiogenesis, play critical roles in tumor progression, invasion, metastasis, and therapeutic resistance. Understanding the complex interactions and mechanisms within the TME is crucial for the development of effective therapeutic strategies in HCC. 

## 3. Emerging Therapeutic Strategies Targeting the HCC TME

### 3.1. Blood Biomarkers and Their Clinical Applications

Blood biomarkers have emerged as promising tools for the early detection and surveillance of HCC. Shahini et al. [23] conducted a critical review of blood biomarkers and their algorithms in HCC diagnosis and surveillance. They emphasized the importance of combining multiple biomarkers, such as alpha-fetoprotein (AFP), a protein induced by vitamin K absence or antagonist-II (PIVKA-II), and Golgi protein 73 (GP73), to improve diagnostic accuracy. The integration of these biomarkers with imaging modalities and clinical data could enhance the prediction of HCC recurrence and overall survival [24].

### 3.2. Immune Checkpoint Inhibitors (ICIs)

ICIs have revolutionized cancer therapy by blocking inhibitory receptors on immune cells, thereby enhancing anti-tumor immune responses. In HCC, ICIs targeting programmed cell death protein 1 (PD-1), programmed cell death ligand 1 (PD-L1), and cytotoxic T lymphocyte-associated protein 4 (CTLA-4) have demonstrated promising clinical activity. Nivolumab stands out in this regard as a fully human IgG4 monoclonal antibody (mAb) that inhibits PD-1 and increases the activity of effector T-cells, allowing them to recognize and destroy cancer cells in the HCC microenvironment [25]. Another anti-PD-1 mAb that has been studied as a second-line treatment in HCC patients is pembrolizumab [26]. Camrelizumab is an anti-PD-1 mAb that binds to a different epitope than nivolumab and pembrolizumab. These findings imply that camrelizumab could be used as a new second-line therapy for individuals with advanced HCC [27]. Anti-PD-L1 mAbs in development include durvalumab, atezolizumab, and avelumab [28]. Tremelimumab is the first anti-CTLA-4 fully human IgG2 mAb approved for the treatment of HCC [29]. In addition to the attention dedicated to the toxicity profile [30], Leone et al. [7] summarized the evolving role of ICIs in HCC treatment and highlighted the potential of combining ICIs with other therapeutic modalities, such as targeted therapies and locoregional treatments, to overcome resistance and improve clinical outcomes [31]. Furthermore, several trials are evaluating ICIs as an adjuvant therapy in combination with TACE and radiofrequency ablation (RFA) because standard locoregional therapies cause the production of neoantigens and local inflammatory factors. According to early findings, this combined approach encourages anti-tumor T-cell response and reduces Treg [32]. In non-randomized research, radiofrequency ablation (RFA), transcatheter arterial chemoembolization (TACE), and chemoablation (CA) enhanced the effectiveness of the ICI tremelimumab (anti-CTLA-4 mAb) in patients with advanced-stage HCC by inducing a CD8 T-cell response [33].

In addition, one trial discovered that atezolizumab plus bevacizumab (anti-VEGF) increased PFS and decreased the risk of progression or mortality compared to atezolizumab alone [34]. In another study, lenvatinib (anti-VEGFR mAb) was combined with pembrolizumab (anti-PD-1 mAb) in patients with unresectable HCC. Due to an increase in anti-tumor activity, patients had a minimal tolerability profile and a high response rate. The FDA considers this drug “revolutionary” for the first-line treatment of patients with unresectable HCC who are ineligible for other therapies [35].

### 3.3. Second-Line Treatments

Patients with advanced HCC who progress or are intolerant to first-line therapy may benefit from second-line treatments; these include two TKIs, cabozantinib and regorafenib, one anti-VEGF monoclonal antibody, ramucirumab, and three ICIs, nivolumab, pembrolizumab, and ipilimumab [36]. A systematic review and Bayesian network meta-analysis by Solimando et al. [6] evaluated the efficacy and safety of various second-line treatments in advanced HCC. The analysis demonstrated that regorafenib, cabozantinib, and ramucirumab significantly improved overall survival compared to the placebo. In that respect, Cabozantinib inhibits tyrosine kinases, including the VEGF receptors 1, 2, and 3, MET, and AXL, which are involved in the progression of hepatocellular carcinoma and the development of resistance to sorafenib, which is the conventional first-line treatment for advanced HCC [37]. Regorafenib, a small-molecule kinase inhibitor, is the first drug approved to treat hepatocellular carcinoma (HCC) in patients who progressed during or after sorafenib therapy [38]. Ramucirumab, an anti-VEGFR2, demonstrated persistent and significant clinical activity in patients with advanced HCC and AFP 400 ng/mL [39]. A further investigation into the combination of these agents with immunotherapies or other targeted therapies is warranted to enhance treatment outcomes. For example, a combination of cabozantinib and atezolizumab may be an effective treatment for some patients with advanced hepatocellular carcinoma, although more research is necessary [40].

### 3.4. BRAF-Targeted Therapies

Although BRAF mutations are rare in HCC, their oncogenic role warrants further investigation into the potential of BRAF-targeted therapies. For instance, NVP-AAL881 is a small molecule inhibitor of RAF-1 and VEGFR2. NVP-AAL881 inhibits the MEK/ERK pathway and the phosphorylation of the signal transducer and activator of transcription 3 (STAT3). Lang et al. demonstrated how the treatment of NVP-AAL881 inhibited the formation of HCC xenograft tumors. Clinical data are required to confirm this oral RAF inhibitor’s potential role in advanced HCC patients [41].

Furthermore, Wenhong Wang et al. demonstrated that magnolin, an active constituent in the volatile oil of Magnolia fargesii, when combined with the BRAF inhibitor SB590885, reduces the growth of HCC cells synergistically [42].

In a preclinical study, Breunig et al. compared sorafenib to the BRAFV600E mutation-specific inhibitor PLX4720 in HCC cell lines for the inhibition of MAPK and PI3K. They found that BRAF and MEK inhibitors have dose-dependent antiproliferative and proapoptotic actions [42]. Gnoni et al. [11] reviewed the role of BRAF in HCC and provided a rationale for future targeted cancer therapies. They suggested that a better understanding of the molecular mechanisms underlying BRAF activation in HCC could lead to the development of novel targeted therapies and personalized treatment strategies.

## 4. Implications of the Barcelona Clinic Liver Cancer (BCLC) Staging and Classification System

The Barcelona Clinic Liver Cancer (BCLC) staging and classification system is widely used in the management of HCC to guide treatment decisions and predict prognosis. This system takes into account tumor characteristics, liver function, and performance status to stratify patients into different stages (0 to D) and assign appropriate treatment strategies [3]. 

Patients with early-stage HCC, including those with a single tumor of less than 2 cm (Stage 0) or a solitary tumor of less than 5 cm or up to three nodules each less than 3 cm (Stage A), are generally candidates for potentially curative therapies, such as surgical resection, liver transplantation, or local ablation treatment modalities that aim to remove or destroy the tumor while preserving liver function [2].

Intermediate-stage patients (Stage B) have multinodular HCC without vascular invasion or extrahepatic spread. Transarterial chemoembolization (TACE) is often recommended for these patients. TACE combines the delivery of chemotherapeutic agents directly into the tumor-feeding arteries with the obstruction of blood supply to the tumor, leading to tumor shrinkage and improved survival [4]. TACE has demonstrated efficacy in controlling tumor growth and extending survival in intermediate-stage HCC patients.

Advanced-stage HCC (Stage C) is characterized by vascular invasion or an extrahepatic spread. Systemic therapies, such as molecular-targeted therapies or immunotherapy, are commonly considered for these patients. Sorafenib, a multikinase inhibitor, is the first-line systemic therapy approved for advanced HCC and has shown modest survival benefits [4]. Lenvatinib, as another targeted therapy, has also been approved as a first-line treatment option for unresectable HCC [5].

The BCLC system also considers liver function, as assessed by the Child–Pugh classification, in treatment decision making. Patients with a well-preserved liver function (Child–Pugh A) are more likely to tolerate aggressive therapies, while those with compromised liver function (Child–Pugh B or C) may require alternative treatment approaches, such as best supportive care or palliative treatments [43].

**Figure 2:**
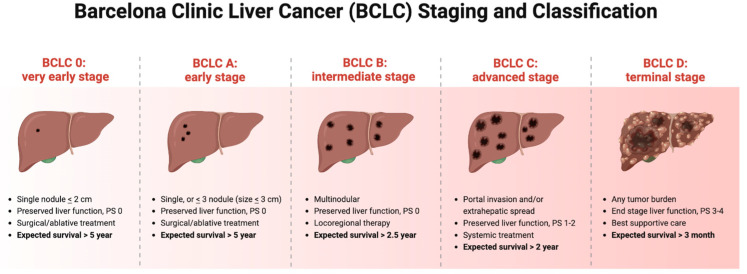
The figure illustrates the BCLC staging and classification system for hepatocellular carcinoma (HCC). This widely accepted system stratifies HCC patients into different stages based on tumor characteristics, liver function, and pathologic stage (PS). The BCLC classification provides a comprehensive framework for guiding treatment decisions, prognosis assessment, and survival outcomes in HCC. The stages range from early-stage tumors amenable to curative treatments (Stage 0–A) to advanced-stage disease requiring systemic therapy (Stage C). The BCLC staging system serves as a valuable tool in clinical practice and clinical trial design, aiding in the management and evaluation of HCC patients. Created with BioRender.com, Publication license n. TN25KYSTLY.

The BCLC staging system has been extensively validated and has demonstrated its utility in predicting survival outcomes and guiding treatment selection in HCC. Several studies have reported a strong correlation between the BCLC stage and overall survival, with more advanced stages associated with poorer prognosis [2,44,45]. Additionally, the BCLC system has been incorporated into clinical guidelines and recommendations for the management of HCC by major international organizations [3,46,47]. (Figure 2).

However, it is important to acknowledge the limitations of the BCLC staging and classification system. While it provides a valuable framework for treatment stratification, it may not fully capture the heterogeneity of HCC. The BCLC system primarily focuses on tumor stage and liver function and does not consider molecular subtypes or genetic alterations that can impact prognosis and treatment response. Efforts are underway to integrate molecular profiling and genetic information into the BCLC system to improve its prognostic accuracy to ensure the individualization of treatment [2].

## 5. Conclusions

A comprehensive understanding of the HCC TME and the implementation of emerging therapeutic strategies are key to advancing the management of this challenging disease. An important role in terms of therapeutic options is associated with liver transplantation. Indeed, Legaz et al. [48] highlighted the importance of Killer cell immunoglobulin-like receptors (KIR) and KIR ligand-dependent alloreactivity in late liver allograft outcomes, suggesting that each KIR+ cell is “encouraged” to sense the missing ligand. Each aKIR+ cell binds putative ligands on allogeneic cells. Therefore, increased cytotoxicity and NK or T-cell activation contribute to an inflammatory environment that favors short-term liver allograft damage. Furthermore, the results of Legaz et al. [48] demonstrate that categorizing transplants via KIR/Class I Human Leukocyte Antigens (HLA-I) ligand matches rather than entire cohorts allow for a more accurate assessment of long-term liver allograft damage because components mediating contradictory effects are hidden in the whole analysis. In conclusion, further research is necessary to identify predictive and prognostic biomarkers, refine therapeutic combinations, and overcome resistance mechanisms to enhance the efficacy of these novel interventions in HCC treatment. By targeting the TME and integrating the BCLC staging system, we can strive for improved patient outcomes and move closer to better managing HCC on a global scale. 

## Data Availability

Not applicable.
